# Neonatal diet impacts liver mitochondrial bioenergetics in piglets fed formula or human milk

**DOI:** 10.1186/s40795-020-00338-7

**Published:** 2020-04-15

**Authors:** Eugenia Carvalho, Sean H. Adams, Elisabet Børsheim, Michael L. Blackburn, Kikumi D. Ono-Moore, Matthew Cotter, Anne K. Bowlin, Laxmi Yeruva

**Affiliations:** 1 Department of Geriatrics, University of Arkansas for Medical Sciences (UAMS), Little Rock, USA; 2 Arkansas Children’s Research Institute, Little Rock, AR USA; 3 Center for Neuroscience and Cell Biology, University of Coimbra, Coimbra, Portugal; 4 Department of Pediatrics, University of Arkansas for Medical Sciences (UAMS), Little Rock, USA; 5 Arkansas Children’s Nutrition Center, 15 Children’s Way, Little Rock, AR 72202 USA

**Keywords:** Human milk, Formula diet, Mitochondria, Liver, Gastrointestinal tract

## Abstract

**Background:**

Neonatal diet impacts many physiological systems and can modify risk for developing metabolic disease and obesity later in life. Less well studied is the effect of postnatal diet (e.g., comparing human milk (HM) or milk formula (MF) feeding) on mitochondrial bioenergetics. Such effects may be most profound in splanchnic tissues that would have early exposure to diet-associated or gut microbe-derived factors.

**Methods:**

To address this question, we measured ileal and liver mitochondrial bioenergetics phenotypes in male piglets fed with HM or MF from day 2 to day 21 age. Ileal and liver tissue were processed for mitochondrial respiration (substrate only [pyruvate, malate, glutamate], substrate + ADP, and proton “leak” post-oligomycin; measured by Oroboros methods), mitochondrial DNA (mtDNA) and metabolically-relevant gene expression analyses.

**Results:**

No differences between the diet groups were observed in mitochondrial bioenergetics indices in ileal tissue. In contrast, ADP-dependent liver Complex I-linked OXPHOS capacity and Complex I + II-linked OXPHOS capacity were significantly higher in MF animals relative to HM fed piglets. Interestingly, p53, Trap1, and Pparβ transcript abundances were higher in MF-fed relative to HM-fed piglets in the liver. Mitochondrial DNA copy numbers (normalized to nuclear DNA) were similar within-tissue regardless of postnatal diet, and were ~ 2–3 times higher in liver vs. ileal tissue.

**Conclusion:**

While mechanisms remain to be identified, the data indicate that neonatal diet can significantly impact liver mitochondrial bioenergetics phenotypes, even in the absence of a change in mtDNA abundance. Since permeabilized liver mitochondrial respiration was increased in MF piglets only in the presence of ADP, it suggests that formula feeding led to a higher ATP turnover. Specific mechanisms and signals involved with neonatal diet-associated differences in liver bioenergetics remain to be elucidated.

## Background

Breastfeeding has been shown to have positive impact on the body’s physiological systems, including the immune system and metabolically-important tissues such as liver, adipose, and cognitive centers in the brain [1, 2]. Recent studies have reported that milk formula-fed (MF) infants present more rapid weight gain during the first weeks of life compared to breastfed infants, and this appears to be associated with weight gain later in life [3–5]. The nutrient composition of human milk (HM) in comparison to milk formulas may play a significant role in the observed metabolic outcomes and the reported health differences when comparing these two neonatal diets [6–11]. Mitochondrial function and energy homeostasis impinge on all of these systems, but the role of infant diet and programming of cellular bioenergetics remains largely unexplored.

A previous study in 8 week old rats fed with HM or donkey milk (DM) in comparison to cow’s milk for 4 weeks showed altered liver and skeletal muscle mitochondrial function and metabolism [12, 13]. Specifically, in isolated liver mitochondria, HM and DM-treated groups had increased succinate or palmitoylcarnitine-associated proton leak, increased states 3 and 4 respiration, and higher mitochondrial markers (e.g., carnitine palmitoyltransferase and citrate synthase activities) when compared to mitochondria from untreated control animals [12]. Mitochondria from animals fed cow’s milk shared some similarities to HM and DM, but the phenotype was complicated by significantly higher adiposity. In muscle mitochondria, leak and states 3 and 4 respiration were again higher in samples derived from HM or DM fed rats vs. untreated control rats, whereas these parameters were equivalent in cow’s milk fed and untreated control rats [13]. In addition, HM and DM increased liver and muscle levels of N-oleoylethanolamine (OEA), a regulator of lipid metabolism [13]. The authors speculated that this could contribute to burning of fat and protect the animals from developing certain obesity-associated metabolic and inflammatory sequelae. Furthermore, they speculated that diet-associated changes in microbiota and increased short chain fatty acid (butyrate) in HM- and DM-fed animals contributed to the differences in metabolism and mitochondrial function through as-yet unknown signaling pathways [12]. We have reported significant alterations in the bioregional gut microbiome when comparing HM- and MF-fed piglets [14], and several studies have indicated differences in gut microbiota comparing breastfed to formula-fed infants [15–18].

There is increasing appreciation for the impact of early-life diet on “programming” of physiological systems with potential metabolic consequences in childhood or adulthood. More important, there is growing consensus that nutritional “programming effects” persist and influence risk for allergies, asthma, obesity, diabetes, and cardiovascular disease later in life [1, 19–24]. We hypothesized that neonatal diet (HM and MF) would differentially affect mitochondrial respiration in the small intestine (ileum) and liver. To address our hypothesis, we used HM and MF-fed piglets under controlled experimental conditions, due to inherent variability associated with sow-fed piglets (e.g., farm housing, mother’s skin contact, and suckling diet). Tissues were collected from piglets fed HM and MF between postnatal day 2–21, to determine substrate- and ADP-driven respiration as well as mitochondrial DNA copy number. To our knowledge, this is the first study to characterize the impact of postnatal feeding paradigms on tissue mitochondrial function in a larger animal model.

## Methods

### Study design

Animals were maintained in accordance with the ethical guidelines for animal research established and approved by the Institutional Animal Care and Use Committee at the University of Arkansas for Medical Sciences. Animals were obtained from Metz Farm (Russellville, AR) that are approved for research. Two-day old White Dutch Landrace Duroc male piglets (Metz Farm, Russellville, AR) were transferred to individual housing at the vivarium in Arkansas Children’s Nutrition Center (ACNC). The study protocol and information on this cohort of animals has been published previously [14]. Piglets were obtained from 30 different sows and housed individually. Based on weights piglets were randomized using excel sheet formula to consume either human milk (HM, *n* = 26) or isocaloric dairy milk-based formula diet (MF; *n* = 26). HM was obtained from the Mothers’ Milk Bank of North Texas (Benbrook, TX). For the MF diet, Similac Advance powder was obtained from Ross Products (Abbott Laboratories, Columbus, OH). Both HM and MF diets were modified to meet the energy and nutrient recommendations of the National Research Council for growing pigs [25]. Diet composition, nutritional contents, and methods related to the feeding paradigm have been published previously [14]. Briefly, with the transition to ACNC, piglets were trained to drink from rubber nipples and were provided 1.047 MJ·kg^− 1^·d^− 1^ of either HM or MF. Piglets were fed with warmed diet every 2 h in the first week of the study, every 4 h in the second week of the study, and every 6 h in the third week of the study through day 21. At day 14, solid “starter pig food” (Teklad diet, TD 140608; Harlan) was slowly introduced until day 21 when 11 randomly selected piglets from each diet group were euthanized. Piglets were euthanized with a mask at an oxygen flow rate of 2.0 LPM and isofluorane mixture of 3–5% at and cardiovascular signs (pulse) were monitored to assure complete unconciousnees prior to exsanguination. The remaining animals were utilized for immunization challenge studies as previously published (*n* = 30) [14], and are therefore not included in the current paper. No significant difference in body weights was observed between HM and MF as previously published [14].

### Tissue processing for mitochondrial functional analysis

Animals were fasted for 8 h prior to tissue collection. A subset of piglets were randomly chosen to assess mitochondrial function (HM, *n* = 8–11; MF, *n* = 8–11; see individual figures). We measured 50 cm from the distal end of small intestine, and at that mark tissue was cut as a 15 cm proximal sample. Ileal and liver samples for analyses of mitochondrial function were processed immediately after collection. A portion of ileum and liver (∼40 mg) was immediately submerged in ice-cold preservation buffer (BIOPS) containing 10 mM Ca-EGTA buffer, 20 mM imidazole, 20 mM taurine, 50 mM K-MES, 0.5 mM dithiothreitol, 6.56 mM MgCl_2_, 5.77 mM ATP, and 15 mM creatine phosphate (pH of 7.1) [26], for mitochondrial respiration analysis within 1–2 h of tissue collection.

Ileal and liver samples were minced (0.1–0.2 mm) using small and sharp forceps while on ice, and chemically permeabilized for 20 min in BIOPS buffer containing 50 μg/ml saponin at 4 °C [27–30]. Samples were transferred to 2 ml of MIR05 buffer (0.5 mM EGTA, 3 mM MgCl_2_, 60 mM K-lactobionate, 20 mM taurine, 10 mM KH_2_PO_4_, 20 mM HEPES, and 110 mM sucrose, and 1 mg/ml essential fatty acid-free bovine serum albumin (Sigma Aldrich, St. Louis, MO, USA; Lot SLBF5061V) followed by 10 min mixing on a shaker to wash away the remaining saponin. Permeabilized tissue explants were blotted on filter paper before being weighed on a precision microbalance. Less than 10 mg of wet weight ileum and about 3 mg of wet weight liver were transferred to an Oxygraph-2 k (O2k) respirometer chamber (Oroboros Instruments, Innsbruck, Austria) containing 2 ml of MIR05 buffer [28, 31, 32].

### High-resolution respirometry (HRR)

Before each experiment, a background calibration was performed on each O2k polygraphic oxygen sensor (POS). This calibration was performed in MIR05 buffer at air saturation. Zero oxygen and instrumental background calibrations were performed at regular intervals throughout the data collection period (∼12 months) using dithionite titrations. This ensured acceptable POS instrumental background and stability over time [26]. Temperature was maintained at 37 ± 0.01 °C by an electronic Peltier during all high-resolution respirometry (HRR) experiments. O_2_ concentration within the MIR05 buffer was recorded at 2 to 4 s intervals from which O_2_ fluxes were calculated in the picomolar range (DatLab version 6; Oroboros Instruments, Innsbruck, Austria) [31]. Once tissue samples had been placed in the O2k chambers, a gas phase was created. About 1 ml of 99% O_2_ was injected into each O2k chamber, and equilibration of the gas phase and MIR05 O_2_ concentrations was monitored until an O_2_ concentration of ∼400 μM was achieved in the MIR05 buffer; O_2_ flux measurements were typically made when O_2_ concentrations were in the range of 200–400 μM to minimize any O_2_ dependency artefacts and to avoid potential limitations in oxygen diffusion in permeabilized tissue samples.

Mitochondrial respiratory capacity and function were evaluated by the sequential addition of substrates and inhibitors. A wide variety of substrate- inhibitor-titration protocols have been used for evaluation of mitochondrial respiration in different samples and under various experimental conditions [30, 33–35]. Here, we evaluated the contribution of complex I and II to mitochondrial respiration, as well as the impact of inhibition of complex V. A bioenergetics protocol was used that informs on specific respiratory pathways converging at the Q-junction of the electron transport chain (Table 1). Effects of NADH-linked substrates (N-pathway), including pyruvate (5 mM), malate (2 mM) and glutamate (PMG, 10 mM) were evaluated to determine non-ATP-linked respiration at Complex I [26]. The present protocol also evaluated oxygen consumption rate (pmol O_2_ · s^− 1^ · mg wet weight tissue^− 1^) to determine the oxidative phosphorylation capacity (“OXPHOS capacity” (*P*)) after adenosine diphosphate (ADP, 5 mM) addition [27, 28, 32]. Subsequently, the “S-pathway” was evaluated using succinate as substrate (10 mM) to determine respiration at Complex II. Also, proton leak respiration at complex I and II was determined by adding oligomycin (OMY; 0.2 μg/ml) after substrates and ADP were provided. The respiratory oxidative capacity results were corrected by the residual oxygen respiration (ROX) obtained after antimycin A (AMA; 12.5 μM) inhibition of electron transport chain Complex III (a.k.a. UQ-cytochrome c oxidoreductase) at the end of the experiment.

**Table 1:** Mitochondrial respiration protocol reagents listed in order of titrations and the corresponding pathway controls and respiratory states

Substrates	Abbreviations	Pathway control/ Respiratory state	Pathway to Q	Explanation
Pyruvate (5 mM), Malate (2 mM), Glutamate (10 mM)	PMG	N/PMG(*L*)	CI	LEAK (*L*) respiration, no adenylates
ADP (5 mM)	ADP	N/PMG(*P*)	CI	OXPHOS capacity (*P*)
Succinate (10 mM)	SUC	NS/PMGS(*P*)	CI + CII	Complex I + II-linked (*P*)
Oligomycin (0.2 μg/ml)	OMY	ROX	ROX	Inhibition of CV
Antimycin A (12.5 μM)	AMA	ROX	ROX	Inhibition of CIII

*Abbreviations:* Adenosine diphosphate (ADP), Complex I (CI), Complex II (CII), LEAK (*L*), NADH- (N) pathway, OXPHOS capacity (*P*), Residual oxygen consumption (ROX)

### Mitochondrial DNA copy number

DNA was extracted from ~ 25 mg of frozen ileal tissue using QIAamp Fast DNA Tissue Kit (QIAGEN, Germantown, MD) following the manufacturer’s protocol. Approximately 500 mg of the liver was homogenized in PBS (Gibco, Thermo Fisher). DNA was extracted from the equivalent of ~ 25 mg of liver using QIAamp Fast DNA Tissue Kit. DNA standards for each target were generated using primers listed in Table 2 to amplify the mitochondrial genes ND1 (519 base pair (bp) amplicon) and Cox1 (477 bp amplicon), and the nuclear gene β-actin (496 bp amplicon) using GoTaq Green Master Mix (Promega, Madison, WI). PCR products were separated on a 1% agarose gel and amplicon bands were excised and extracted using QIAquick Gel Extraction Kit (QIAGEN, Germantown, MD). Concentrations were determined using a NanoDrop 1000 Spectrophotometer (NanoDrop, Wilmington, DE) and a Qubit 2.0 fluorometer using the dsDNA HS Assay Kit (Invitrogen). Copy number per μl of DNA was determined using the equation:

**Table 2:** Primers used for mitochondrial DNA copy number measurement in piglet ileum and liver

Gene	Forward Primer 5′-3’	Reverse Primer 5′-3’	Amplicon (bp)	Use
*Cox1*	CTCTGGGCTTCATCTTCCTATTC	GAGGACATCCGTGTAGTCATTC	477	Standard Curve
*Nd1*	GCCTAGCAGTATACTCTATCCT	CGTATCGGAATCGTGGGTATG	519	Standard Curve
*Actb*	CTGGCATTGTCATGGACTCT	GATCGAGTTGAAGGTGGTCTC	496	Standard Curve
*Cox1*	GTAGTCGCACACTTCCACTATG	TGAGTGTGTACCCGGAGAATA	100	Real-time PCR
*Nd1*	TTATCTCAACCCTAGCAGAAACC	AAAGGTCCGGCTGCATATT	103	Real-time PCR
*Actb*	ACCTGACCGACTACCTCAT	GCAGAGCTTCTCCTTGATGT	101	Real-time PCR

number of copies/μl = (amount (ng/μl) * 6.022 × 10^23^ number /mole) / (length (bp) * 1 × 10^9^ ng/g * 650 g/mole of bp).

Standards were serially diluted 10-fold from 1 X 10^8^ copies/μl to 1 X 10^3^ copies/μl to construct a 6-point standard curve. For real-time PCR, new primers located within the amplicon for ND1, Cox1 and β-actin (Table 2) were designed using Integrated DNA Technology’s (IDT) PrimerQuest Tool (www.idtdna.com/Primerquest/Home/Index). DNA (200 pg) was used in a 10 μl PCR reaction using Fast SYBR Green Master Mix (Applied Biosystems) on a ViiA 7 Real-Time PCR System (Applied Biosystems). mtDNA copy number was calculated using standard curves and normalized using the abundance of the β-actin nuclear gene. All available piglet samples were used to determine mitochondrial DNA copy number (HM, *n* = 11; MF, *n* = 11 per tissue).

### Gene expression

RNA was extracted from ~ 30 mg of frozen ileum tissue using RNeasy Plus Mini Kit (Qiagen) according to manufacturer’s protocol. For liver RNA, ~ 100 mg of frozen livers were homogenized using miRNeasy Mini Kit (Qiagen) and homogenate equivalent to ~ 30 mg of liver from each sample was used for RNA extraction. Concentration was determined using UV absorbance. One μg of total RNA was used to create cDNA using iScript cDNA Synthesis Kit (Bio-Rad, Hercules, CA) according to manufacturer’s protocol. cDNA was diluted and 10 ng of cDNA was used in a PCR reaction using Fast SYBR Green Master Mix (Applied Biosystems) on a ViiA 7 Real-Time PCR System (Applied Biosystems). A standard curve was generated by pooling undiluted cDNA from different treatments to create a master pool. The master cDNA mix was serially diluted 5-fold a total of four times, for an arbitrary five-point standard curve. RNA expression was normalized for the ileum using the geometric mean of three (Rps16, Rpl27, and 18S) reference RNAs and for the liver using the geometric mean of two (Rps16 and 18S) reference RNAs that were not altered by treatment. All available piglet samples were used to determine mitochondrial gene expression (HM, *n* = 11; MF, *n* = 11). One MF fed piglet liver sample was removed due to poor RNA quality (RIN ~ 6.5). Primers were designed using IDT’s PrimerQuest and are listed in Table 3**.**

**Table 3:** Primers used for mitochondrial gene expression assays in piglet ileum and liver

Gene	Forward Primer 5′- 3’	Reverse Primer 5′- 3’
Cycs	CCAAACCTCCATGGTCTCTTT	TACTCCATCAGTGTCTCCTCTC
Hspa9	TCAACAGGAACACCACCATT	TCTCTCTCACCCTGACATACTT
Nrf1	CGATGGCACTGTCTCTCTTATC	CCATCAGCCACAGCAGAATA
p53	CCGAGTACTTGGATGACAGAAA	GTTGTAGTGGATGGTGGTACAG
Pgc1α	CCCACAACTCCTCCTCATAAAG	TCACTGTACCGGGTCTTCT
Pparβ	CATGAAGCTGGAGTACGAGAAG	AGCGAATGGCGTTGTAAGA
Sco2	AAGGACGAGGACCAGGATTA	TGCCGTAGTAGTCGGTGAA
Tfam	GTGGAGGGAACTTCCTGATTC	TGACTTGGAGTTAGCTGTTCTT
Trap1	CAGCTTCGACCACCTCTTC	CCAGCAGCCCATCCTTATT
Uqcr10	GAGGTTGTACTCCCTGTTGTTT	CCTGGTTGATGTGTTCGTAGAT
Rps16	TGATCCTCGTCGCTGTGAATCCAA	ACGCTGATCATCACGATGGGCTTA
Rpl27	AAGTTCATGAAACCCGGGAAGGTG	TCGGTCTGAGGTGCCATCATCAAT
18S	CCTGTAATTGGAATGAGTCCAATTT	ATACGCTATTGGAGCTGGAATTACC

### Statistical analyses

All data are presented as means ± SEM. The data were analyzed by two-tailed t-test and *p* < 0.05 was considered to be statistically significant between the groups.

## Results

### Mitochondrial respiration phenotypes

**Fig. 1:**
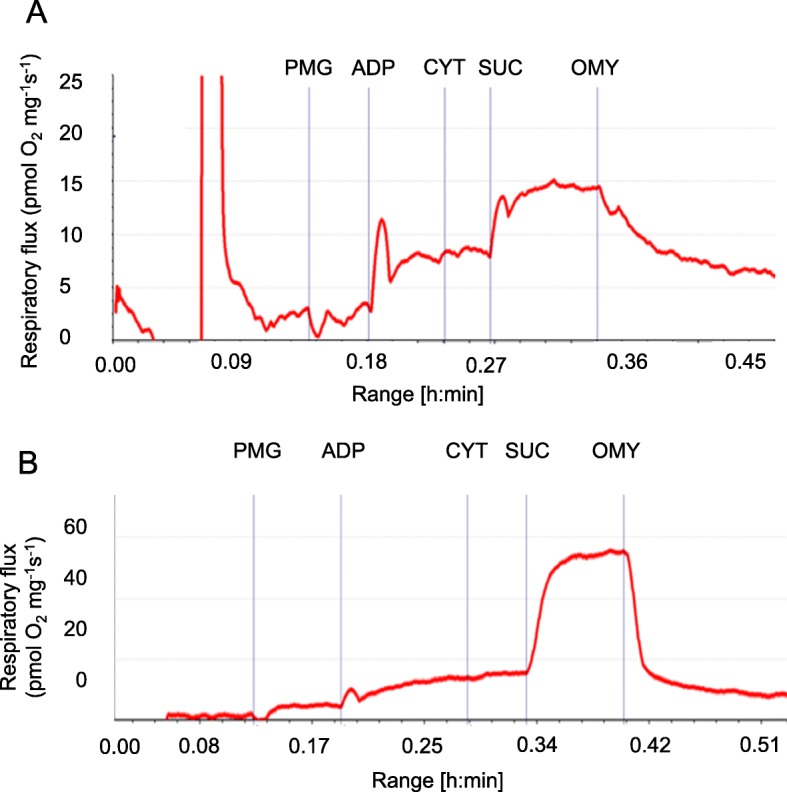
Illustrative respiratory fluxes of permeabilized ileum tissue from 21 day old piglets. **a** ileum and **b** liver sample original trace from the Oroboros oxygraph shows responses to the substrate- inhibitor titration protocol as described in results section

A trace of oxygen flux using permeabilized ileum and liver samples derived from a 21-day old piglet fed with HM is shown in Fig. 1. This protocol provides assessment of “respiratory leak” (*L*) supported primarily by electron flow through complex I of the respiratory chain after addition of pyruvate, malate and glutamate (PMG-*L*). The protocol also provides the OXPHOS capacity (*P*) after ADP is added following the pyruvate, malate and glutamate (PMG-*P*); P represents maximal Complex I-linked ADP-linked OXPHOS capacity through the N-pathway, electron transfer occurs by Complex I. Subsequently, succinate (SUC) is added to induce complex II-linked respiration. We determined the contribution of electron flow from both complex I and II respiration, representing the maximal ADP-linked OXPHOS capacity that was measured in ileum. Next, OMY was added to assess leak respiration when substrates and ADP were provided, but ATP synthase inhibited, measuring proton leak from complex I and II.

**Fig. 2:**
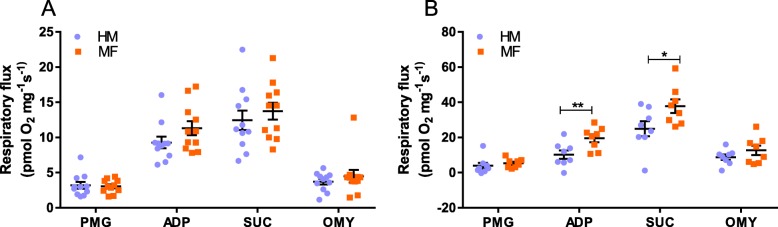
Respiratory fluxes of piglet ileum on day 21, from animals consuming human milk (HM) or cow milk-based formula (MF). **a** Mass-specific respirometry results of permeabilized ileum tissue of 21-day old piglets fed either HM or MF (*n* = 11/diet group). **b** Mass-specific respirometry results of permeabilized liver of 21-day old piglets fed either HM (*n* = 8) or MF (*n* = 8) for a period of 21 days starting on postnatal day 2. PMG: pyruvate, malate and glutamate; ADP: adenosine diphosphate; SUC: succinate; OMY: oligomycin; The data are presented as mean ± SEM. The data were analyzed by 2-tailed t-test and *p < 0.05, **p < 0.01 was considered significant between the diet groups

We assessed the impact of neonatal diets on mitochondrial respiration in permeabilized ileum on day 21 (Fig. 2a). No significant differences were observed in the permeabilized ileum explants for all parameters tested: PMG respiratory leak, Complex I-linked OXPHOS capacity, measured after adding ADP, Complex I- and II-linked OXPHOS capacity (CI + II *P*) measured after addition of succinate (SUC), and Complex I and II-linked leak (OMY). In the liver (Fig. 2b), higher Complex I-linked OXPHOS capacity and higher Complex I- and II-linked OXPHOS capacity were observed in MF-fed piglets compared to HM-fed piglets.

### Mitochondrial DNA

**Fig. 3:**
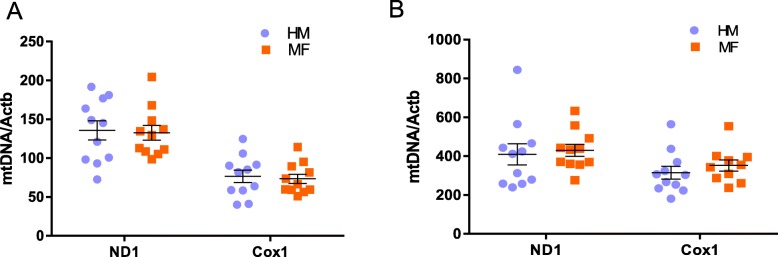
Mitochondrial DNA (mtDNA) copy number in 21 day old piglet ileum and liver tissues. **a** Day 21 mtDNA (*n* = 11/ diet group) of ileum and **b** day 21 mtDNA (*n* = 11/diet group) of liver. mtDNA values are normalized to the nuclear DNA target gene beta-actin. The data are presented as mean ± SEM. The data were analyzed by 2-tailed t-test to determine the significance between the diet groups. HM - Human milk; MF - Cow milk-based formula

Mitochondrial DNA copy number, normalized to nuclear DNA copy number, did not significantly differ on day 21 between the HM and MF diet groups in ileum or liver (Fig. 3**)**. Notably, mtDNA was ~ 2–3 times higher in liver compared to ileum.

### Expression of select genes relevant to metabolism and mitochondrial bioenergetics

**Fig. 4:**
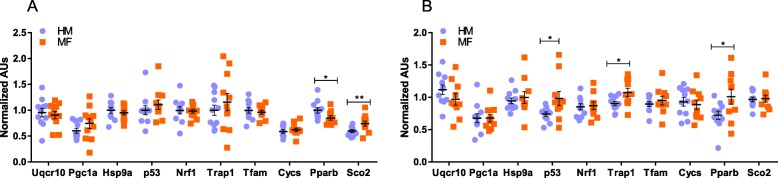
Gene expression analysis of piglet ileum and liver at day 21, from animals consuming human milk (HM) or cow milk-based formula (MF). **a** lleum (*n* = 11/ diet group) and **b** liver (*n* = 10–11/diet group) samples expression of Uqcr10, Pgc1α, Hspa9, p53, Nrf1, Trap1, Tfam, Cycs, Pparβ and Sco2. Normalized arbitrary units were calculated using geometric mean of 2–3 reference genes and standard curves generated for each target (see methods section). The data are presented as mean ± SEM. The data were analyzed by 2-tailed t-test, *p < 0.05, **p < 0.01 was considered significant between the diet groups

We measured gene expression for select transcripts involved in mitochondrial function and metabolism from ileum and liver. In ileum, no significant differences were noted between the diet groups with respect to Ubiquinol-Cytochrome C Reductase (Uqcr10), Peroxisome Proliferator-Activated Receptor Gamma Coactivator 1-Alpha (Pgc1α), Cytochrome C (Cycs), Heat Shock Protein Family A, Member 9 (Hspa9), p53, Nuclear Respiratory Factor 1 (Nrf1), TNF Receptor Associated Protein 1 (Trap1), and Transcription Factor A mitochondria (Tfam) transcript abundances at day 21 (Fig. 4a). Interestingly, in ileum Peroxisome Proliferator-Activated Receptor Beta (Pparβ) expression was significantly lower in MF group at day 21 relative to the HM group, while Cytochrome C Oxidase Assembly Protein (Sco2) expression was significantly higher (Fig. 4a). In liver, p53, Trap1 and Pparβ transcript abundances were higher in MF-fed relative to HM-fed piglets (Fig. 4b).

## Discussion

The current proof-of-principle study focusing on piglet ileal and liver tissue uncovered novel findings with respect to infant diet and tissue mitochondrial function, and suggest that select aspects of liver bioenergetics are altered by neonatal diet.

The area of developmental programming of bioenergetics, which encompasses systems involving the mitochondrial management of fuels and metabolites to ultimately support efficient energy conversion to ATP and regulation of reactive oxygen species generation, remains largely unexplored. It may be expected that one’s diet patterns and macronutrient availability would influence mitochondrial function and adaptations that would ensure proper maintenance and development of bioenergetics systems. This may be especially profound in tissues that are proximal to gut-derived signals. Signals from the gut microbiome, or that are changed coincident with microbiome shifts, reduce liver or muscle expression of some metabolism genes (e.g., *PGC-1α, SIRT1*, and *AMPK*) and regulate mitochondrial biogenesis and function (reviewed in [36]). The most obvious example is butyrate, which can mediate *PGC-1α* induction in rodent muscle. Administration of the probiotic *Lactobacillus rhamnosus* CNCMI-4317 to adult animals appeared to activate PPAR-α pathways coincident with higher oxidative phosphorylation capacity. Several clinical and animal studies have shown gut microbiota differences in breast-fed versus formula-fed neonates [14, 37–41], and other studies have demonstrated that gut microbiota may increase fatty acid uptake and oxidation through secondary bile acids [42–44]. The gut microbes *Bacteroides, Eubacterium,* and *Clostridium* genera degrade the primary bile acids to secondary bile acids [45]. Interestingly, from the same piglets studied herein, we have reported higher abundance of *Bacteroides* in HM-fed piglets relative to MF-fed piglets [14]. In addition, rats fed with HM also showed increased *Bacteroides* colonization compared to control animals along with enhanced mitochondrial activity/proton leakage [12]. Since the GI tract is in direct contact with gut microbes, and other splanchnic tissues such as liver are immediately downstream, mitochondria and mitochondrial regulators in splanchnic tissue cells may be particularly sensitive to microbe-derived signals. Such a hypothesis awaits testing in future studies.

In addition to microbial signals, there is the possibility that food components themselves influence mitochondrial function. Rat models (post-weaned, adult animals) indicate that mitochondrial function of liver and skeletal muscle can be modulated by dietary components [13, 46–51]. However, nothing is known about the effect of postnatal diet on mitochondrial function, especially in the splanchnic tissues that would be exposed to dietary components and microbial-derived factors that might regulate metabolic pathways. For instance, human milk and donkey milk improved glucose and lipid metabolism, and modified mitochondria in adult rat skeletal muscle when compared to untreated control animals [13], although it could not be ascertained if these differences were driven by food components or other factors emanating from the gut microbes or host tissues. Furthermore, diet components have been shown to impact mitochondrial bioenergetics in animal models and cell culture (reviewed in [52]). In liver and muscle there is evidence that exposure to high levels of saturated fatty acids promotes mitochondrial fission-like events, decreased proton leak, and higher ROS, whereas ω3 polyunsaturated fatty acids have the opposite effect in animal models. Whether or not fats and other components of the neonatal diet influence mitochondrial function or programming of mitochondrial bioenergetics remains to be evaluated experimentally. Regardless of specific mechanisms, the current study indicates that HM and MF feeding can elicit different bioenergetics phenotypes in the liver: piglets fed MF displayed significantly higher ADP-linked respiration. The increased permeabilized liver respiration was not due to differences in the number of mitochondria (since copy number per genomic beta-actin was not different between the diet groups), and was not due to gross differences in proton leak. From these observations, it appears that higher ATP turnover in the MF liver drove higher O_2_ consumption. On the other hand, neonatal feeding paradigm did not appear to alter ileum bioenergetics compared to HM fed piglets, highlighting a potential tissue specificity in the diet-splanchnic tissue bioenergetics interplay. The current study design does not allow us to determine the specific mechanisms involved, but one speculation is that in the case of ileum there is more proximal exposure to diet- and microbe-derived tissue regulators, whereas metabolic regulation in the liver is an aggregate of gut-derived and systemic factors. Future studies are needed to validate these findings in other models, and to determine if additional regions of the gut display changes in mitochondrial function in response to neonatal feeding paradigms.

## Conclusion

Our data demonstrate that neonatal diet impacts liver mitochondrial bioenergetics phenotypes. In addition, in the presence of ADP, mitochondrial respiration was increased in MF piglets relative to HM-fed piglets, suggesting that formula feeding led to a higher innate hepatic ATP turnover. Future studies are needed to understand how diet-associated differences in gut tissue mitochondrial respiration come about, and if there are potential long-term ramifications with respect to energy homeostasis, oxidative stress, and other outcomes. Clearly, more work needs to be done to evaluate this working model of neonatal diet effects on mitochondrial function. The specific diet- or microbe-derived signals, and/or host signals, that regulate mitochondrial activities (and their temporal relationship to the neonatal period), as well as specific gut regions impacted, remain to be identified. Should infant diet effects on bioenergetics described herein recapitulate in human tissues, it would have profound implications in terms of understanding fundamental molecular events that differentiate physiological responses to formula-feeding and breastfeeding.

## Data Availability

All generated or analyzed data are included in the article. The datasets used and/or analyzed during the current study are available from the corresponding author on reasonable request.

## Abbreviations

ACNC: Arkansas Children’s Nutrition Center
ADP: Adenosine diphosphate
AMA: Antimycin A
DM: Donkey milk
HM: Human milk
HRR: High-resolution respirometry
OEA: Oleoylethanolamine
OMY: Oligomycin
PMG-L: Pyruvate, malate and glutamate
SUC: Succinate
